# Prognostic Role and Determinants of Ascending Aorta Dilatation in Non-Advanced Idiopathic Pulmonary Fibrosis: A Preliminary Observation from a Tertiary University Center

**DOI:** 10.3390/jcm14041300

**Published:** 2025-02-15

**Authors:** Andrea Sonaglioni, Antonella Caminati, Greta Behring, Gian Luigi Nicolosi, Gaetana Anna Rispoli, Maurizio Zompatori, Michele Lombardo, Sergio Harari

**Affiliations:** 1 Division of Cardiology, MultiMedica IRCCS, 20123 Milan, Italy; michele.lombardo@multimedica.it; 2 Semi-Intensive Care Unit, Division of Pneumology, MultiMedica IRCCS, 20123 Milan, Italy; antonella.caminati@multimedica.it (A.C.); greta.behring@unimi.it (G.B.); sergio.harari@unimi.it (S.H.); 3 Division of Cardiology, Policlinico San Giorgio, 33170 Pordenone, Italy; gianluigi.nicolosi@gmail.com; 4 Division of Radiology, MultiMedica IRCCS, 20123 Milan, Italy; gaetanaanna.rispoli@multimedica.it; 5 DIMES Department, University of Bologna, 40126 Bologna, Italy; maurizio.zompatori@unibo.it; 6 Department of Clinical Sciences and Community Health, Università di Milano, 20122 Milan, Italy

**Keywords:** idiopathic pulmonary fibrosis, ascending aorta diameter, ascending aorta dilatation, mortality, outcome

## Abstract

**Background:** Patients with idiopathic pulmonary fibrosis (IPF) have a high prevalence of cardiovascular (CV) risk factors and an increased CV disease burden. The aim of this study was to investigate the prognostic role of the ascending aorta (AA) diameter in patients with mild-to-moderate IPF and to identify the main determinants of AA dilatation. **Methods:** All IPF patients without severe pulmonary hypertension who underwent a multi-instrumental evaluation, comprehensive of high-resolution computed tomography (HRCT) and transthoracic echocardiography (TTE), between September 2017 and November 2023, were retrospectively analyzed. The primary endpoint was the composite of “all-cause mortality or re-hospitalization for all causes”, over a medium-term follow-up. The secondary endpoint was to evaluate the independent predictors of AA dilatation. Additionally, Bland–Altman analysis was used to assess the accuracy and precision of echocardiography-derived AA diameters compared with non-ECG gated HRCT measurements. **Results:** A total of 105 IPF patients and 102 age-, sex-, and CV risk factor-matched controls without IPF were evaluated retrospectively. Over a follow-up of 3.9 ± 1.9 yrs, 31 patients died and 47 were re-hospitalized. AA/height (HR 1.15, 95% CI 1.06–1.25, *p* < 0.001) was independently associated with the primary endpoint, whereas unindexed AA (HR 1.01, 95% CI 0.96–1.06, *p* = 0.83) and AA/BSA (HR 1.00, 95% CI 0.89–1.11, *p* = 0.39) were not. An AA/height > 20 mm/m showed 100% sensitivity and 63% specificity (AUC = 0.78) for predicting the primary endpoint. C-reactive protein (OR 1.87; 95% CI 1.21–2.89, *p* = 0.005) and left ventricular mass index (OR 1.13, 95% CI 1.04–1.24, *p* = 0.006) were independently associated with an AA/height > 20 mm/m in the whole study group. The Bland–Altman analysis revealed a bias of +2.51 mm (with the 95% limits of agreement ranging from −3.62 to 8.65 mm) for AA estimation, suggesting a general overestimation of the AA diameter by TTE in comparison to HRCT. **Conclusions:** AA dilatation is predictive of poor outcomes in IPF patients without advanced lung disease over a mid-term follow-up. The AA/height assessment may improve the prognostic risk stratification of IPF patients.

## 1. Introduction

Idiopathic pulmonary fibrosis (IPF) is a chronic, progressive, fibrotic interstitial lung disease of unknown etiology, which occurs primarily in older adults [1]. The prognosis of IPF is poor, with an estimated median survival of 2–5 years from the time of diagnosis [2]. Several comorbidities, along with intrinsic and extrinsic risk factors, may contribute to the development of IPF [3,4,5].

The literature data indicate that IPF patients have a high prevalence of cardiovascular (CV) risk factors and increased CV disease burden, including coronary artery disease, heart failure, atrial fibrillation, and cerebrovascular disease [6,7,8]. Moreover, IPF may affect the vasculature beyond the lungs and, through an increased systemic inflammation, also accelerate atherosclerosis and degenerative processes of the arterial walls [9,10], thus potentially contributing to ascending aorta (AA) dilatation.

Recent population studies [11,12,13,14,15,16] have reported that an increased AA diameter is independently associated with a greater risk of CV events in large cohorts of individuals. As far as we know, no previous study was specifically focused on the prognostic role of AA diameters in IPF patients. Considering the high CV risk profile of IPF patients, we hypothesized that the AA diameter might be associated with poor outcome, over a mid-term follow-up period. Accordingly, the present study aimed at primarily investigating the prognostic role of the AA diameter in patients with mild-to-moderate IPF and determining the main parameters independently associated with AA dilatation in the same cohort of patients. The reproducibility of AA measurements by transthoracic echocardiography (TTE), in comparison to those derived from high-resolution computed tomography (HRCT) scans, will be discussed as well.

## 2. Materials and Methods

### 2.1. Study Population

The present study retrospectively analyzed all IPF patients without severe pulmonary hypertension (PH), followed up between September 2017 and November 2023 at the Division of Pneumology of our Institution. All patients with mild-to-moderate IPF who underwent a multi-instrumental evaluation, comprehensive of HRCT, spirometry and diffusing capacity of the lungs for carbon monoxide (DLCO), six-minute walking test (6MWT), electrocardiography (ECG), conventional TTE and carotid ultrasonography, were included. The same cohort of IPF patients was the object of previous investigations focusing on left atrial strain evaluation [17], sonographic examination of the carotid arteries [18], arterial elastance assessment [19], and finally on the prognostic role of the CHA_2_DS_2_-VASc score [20]. This retrospective cohort of IPF patients was compared with healthy individuals without IPF, matched by age, sex, and cardiovascular risk factors [21]. The control group was selected from the Outpatient Cardiology Division of our Institution.

IPF was defined according to the 2022 ATS/ERS/JRS/ALAT Clinical Practice Guidelines [1].

The inclusion criteria were: (1) mild-to-moderate IPF, defined by forced vital capacity (FVC) > 50%, DLCO > 35% and TTE-derived tricuspid regurgitation velocity (TRV) < 3.4 m/s [22]; (2) IPF diagnostic work-up comprehensive of blood tests, spirometry and DLCO, 6MWT, HRCT, ECG, TTE, and carotid ultrasonography; (3) hemodynamic stability.

The exclusion criteria were: (1) severe pulmonary hypertension [22] and/or congestive right heart failure at basal evaluation; (2) hemodynamic instability at basal evaluation; and (3) incomplete laboratory and instrumental data.

The following information was obtained from the patients’ medical records: demographics and anthropometrics; prevalence of relevant cardiovascular risk factors; history of cardiovascular and/or cerebrovascular events; ECG data; main comorbidities; blood tests comprehensive of serum levels of C-reactive protein (CRP) and estimated glomerular filtration rate (eGFR) [23]; and finally, the medical treatment during the hospitalization.

Each IPF patient included in the study underwent anamnesis, objective examination, HRCT, spirometry and DLCO, 6MWT, ECG, TTE, and carotid ultrasonography, on the same day.

The study protocol was authorized by the Comitato Etico Territoriale Lombardia 5 (Committee’s reference number 508/24, date of approval 22 October 2024).

### 2.2. High-Resolution Computed Tomography

A HRCT was obtained at the time of diagnosis in all IPF patients. Based on the HRCT characteristics, two expert radiologists (M.Z. and G.A.R.) classified each IPF patient into a definite usual interstitial pneumonia (UIP) pattern (subpleural and basal predominant honeycombing with or without traction bronchiectasis), a probable UIP pattern (subpleural and basal predominant reticular pattern with traction bronchiectasis or bronchiolectasis), or an indeterminate UIP pattern (peribronchovascular and subpleural ground-glass opacities, intermingled with fine reticulation but no honeycombing or traction bronchiectasis). The two radiologists also estimated the coronary artery calcification (CAC) score according to the Agatston method [24] by using semiautomatic software (CaScoring, Syngo.via VB30A, Siemens Healthineers). Coronary calcium was classified using a threshold of 130 Hounsfield units (HU) [25]. Internal diameters of the aortic root and AA were measured at end-expiration, without ECG-gating.

### 2.3. Conventional Transthoracic Echocardiography

All echocardiographic examinations were performed by using a Philips Sparq ultrasound machine (Philips, Andover, MA, USA) with a 2.5 MHz transducer.

The following M-mode and 2D echocardiographic parameters were recorded: relative wall thickness (RWT); left ventricular mass index (LVMi); left ventricular end-diastolic volume index, left ventricular end-systolic volume index, and left ventricular ejection fraction (LVEF), estimated with the biplane modified Simpson’s method [26]; left atrial volume index; right ventricular inflow tract (RVIT), right ventricular (RV) to left ventricular (LV) basal diameter ratio, and tricuspid annular plane systolic excursion (TAPSE) from the apical four-chamber view; finally, the inferior vena cava (IVC) transverse diameter.

Doppler measurements included the E/A ratio and the average E/e’ ratio, as indices of LV diastolic function and left ventricular filling pressures (LVFPs), respectively [27]. Systolic pulmonary artery pressure (sPAP) was derived by the modified Bernoulli equation [22]. The ratio between TAPSE and sPAP was measured as a noninvasive index of RV/pulmonary artery (PA) coupling [28].

The degree of valvulopathy was assessed according to the AHA/ACC recommendations [29].

Finally, aortic root and AA diameters were measured at end-diastole from the parasternal long-axis view, using the “leading edge-to-leading edge” convention, as recommended by the latest ESC guidelines [30]. The values of the aortic root and AA diameters were reported as unindexed and indexed to body surface area (BSA) and height.

### 2.4. Endpoint Definition

The primary endpoint of this study was to identify the independent predictors of the composite of “all-cause mortality or re-hospitalization for all causes” in a retrospective cohort of IPF patients without severe PH, over a medium-term follow-up.

The secondary endpoint was to evaluate the independent predictors of an increased AA diameter, whose cut-off value was statistically determined, in the same study group.

The causes of death and/or rehospitalizations were investigated, for each IPF patient, through the review of the medical records available in the hospital archive and/or telephone interviews.

### 2.5. Statistical Analysis

For statistical power calculation, we hypothesized that, by dividing IPF patients into two main categories (those with an AA diameter/BSA > 19 mm/m^2^ and those with an AA diameter/BSA ≤ 19 mm/m^2^), IPF patients with AA dilatation might have a significantly higher risk of “all-cause mortality or re-hospitalization for all causes” than those with normal AA size, over a mid-term follow-up period. Assuming that IPF patients with an AA diameter/BSA > 19 mm/m^2^ and those with an AA diameter/BSA ≤ 19 mm/m^2^ might have a 3-year “all-cause mortality or re-hospitalization for all causes” of 20% and 10%, respectively, a sample size of 105 IPF patients would reach a statistical power of 100% for determining a statistically significant difference in the rates of “all-cause mortality or re-hospitalization for all causes” between the two groups of individuals, using a two-tailed *t*-test with a type I error at 5%.

Given that all data were determined to be normally distributed, continuous data were summarized as mean ± standard deviation, while categorical data were presented as numbers (percentage). The difference between the means was estimated using the *t*-test, whereas categorical variables were compared using the Chi-square test.

Univariate and multivariate Cox regression analyses were performed to identify the independent predictors of the composite of “all-cause mortality or re-hospitalization for all causes” in IPF patients, over a medium-term follow-up. The following variables were included in the Cox regression analysis: age and male sex (as demographics), CRP (as a systemic marker of inflammation), FVC (as a measurement of pulmonary function), definite UIP pattern (as HRCT subtype of IPF), LVEF (as an index of LV systolic function), TAPSE/sPAP ratio (as an index of RV-PA coupling), unindexed and/or indexed AA diameters, CAC score (as an index of atherosclerotic burden), and, finally, beta-blocker treatment. For each variable investigated, correspondent hazard ratios with 95% confidence intervals (CIs) were calculated.

The receiver operating characteristics (ROC) curve analysis was performed to establish the sensitivity and specificity of the main statistically significant continuous variables for predicting the primary endpoint. The area under the curve (AUC) was estimated.

Kaplan–Meier survival curves were designed to measure the differences between the AA diameter categories in the rates of “all-cause mortality or rehospitalizations for all causes”, over a mid-term follow-up, for the whole cohort of IPF patients. The survival curves were compared using the log-rank test.

Univariate and multivariate logistic regression analyses were performed for detecting the independent predictors of AA dilatation, whose optimal cut-off was determined using the Youden Index, in IPF patients.

Finally, Bland–Altman analysis [31] was used to assess the accuracy and precision of echocardiography-derived AA diameters compared with non-ECG gated HRCT measurements. The accuracy of echocardiography was assessed by estimating the mean difference between noninvasive and invasive measures of the AA diameters and their 95% confidence interval (CI). Precision was assessed by calculating the lower and upper limits of agreement between noninvasive and invasive measures of the AA diameters.

Statistical analysis was performed using SPSS software version 28 (SPSS Inc., Chicago, IL, USA).

## 3. Results

### 3.1. Clinical Findings

A total of 105 IPF patients and 102 age-, sex-, and cardiovascular risk factor-matched controls without IPF were evaluated retrospectively.

The two study groups showed a moderate-to-high prevalence of smoking history and a moderate prevalence of hypertension, type 2 diabetes mellitus, and dyslipidemia. Compared to controls, IPF patients had a significantly higher atherosclerotic burden, as expressed by the greater prevalence of ≥50% carotid artery stenosis, CAC on HRCT, lower extremity peripheral artery disease, and finally polidistrectual vasculopathy; however, IPF patients were not found to have a significantly increased prevalence of coronary artery disease and/or cerebrovascular events compared to controls. The two study groups showed a similar prevalence of non-cardiovascular comorbidities, such as cancers, chronic obstructive pulmonary disease (COPD), obstructive sleep apnea syndrome (OSAS), gastroesophageal reflux disease (GERD), hypothyroidism and mixed anxiety–depressive disorder. Analysis of blood tests revealed that serum levels of CRP were significantly increased in IFP patients compared to controls (1.7 ± 2.7 vs. 0.9 ± 2.1 mg/dL, *p* = 0.02), whereas no statistically significant differences were observed between the two groups of patients with regard to serum hemoglobin, eGFR glucose, N-terminal pro-B-type natriuretic peptide (NT-proBNP), and low-density lipoprotein (LDL) cholesterol. A cardioprotective treatment comprehensive of beta blockers and statins was more commonly prescribed to controls, whereas approximately half of the IPF patients were treated with oxygen therapy and anti-fibrotic agents (Table 1).

### 3.2. Instrumental Findings

Table 2 summarizes all the relevant instrumental parameters measured in the two study groups.

On HRCT, 60% of IPF patients were diagnosed with a “definite” UIP pattern, 24.8% with a “probable” UIP pattern, and the remaining 15.2% with an “indeterminate” pattern; moreover, an elevated CAC score (698.9 ± 879.8 HU) was obtained. Pulmonary function tests (PFTs) showed a mild reduction in FVC%, forced expiratory volume in 1 s (FEV1)%, and total lung capacity (TLC)%, whereas DLCO% was moderately impaired. The 6 min distance walked by IPF patients was slightly reduced in comparison to the normal range for healthy individuals (between 400 and 700 m) [32]. No statistically significant differences were observed between the two study groups in heart rate, atrial fibrillation prevalence, and intraventricular delay on resting ECG. On TTE, left-sided cardiac chamber cavity size did not differ between IPF patients and controls. The most common LV geometric pattern detected in both groups of patients was LV concentric remodeling with normal LV systolic function and first-degree diastolic dysfunction. However, LVFPs, as expressed by the E/average e’ ratio (14.0 ± 4.5 vs. 11.9 ± 4.9, *p* = 0.001), were significantly higher in IPF patients than controls. No significant valvulopathy was reported in the two study groups. IPF patients were found with a mild degree of RV enlargement, as quantified by both RVIT diameter and RV/LV basal diameter ratio measured from the apical four-chamber view. Nevertheless, RV systolic function, assessed by the M-mode derived TAPSE (22.0 ± 4.7 vs. 22.9 ± 3.7 mm, *p* = 0.13) was normal and similar in both groups of patients. The echocardiographic evaluation of pulmonary hemodynamics revealed significantly increased values of TRV, IVC diameter, and sPAP in IPF patients compared to controls, compatible with mild-to-moderate PH. A concomitant moderate impairment in TAPSE/sPAP ratio was observed in IPF patients in comparison to controls and to the accepted normal range, which is typically between 0.8 and 1.8 [28]. Concerning the assessment of the thoracic aorta, the aortic root and AA diameters indexed to BSA were similar in the two study groups. The majority of IPF patients (74.3% in total) were found with increased AA diameters indexed to BSA, in comparison to the accepted reference values [26]. Compared to controls, IPF patients were diagnosed with significantly larger unindexed aortic root and AA diameters and significantly larger aortic root and AA diameters indexed to height.

### 3.3. Survival Analysis

The mean follow-up time was 3.9 ± 1.9 yrs. During the follow-up period, 31 IPF patients died and 47 were re-hospitalized due to: (1) CV causes (21.3%): acute ischemic stroke/transient ischemic attack (6 patients) and acute coronary syndrome (4 patients); (2) cardiopulmonary causes (42.5%): right heart failure (12 patients) and acute pulmonary embolism (8 patients); (3) pulmonary causes (36.2%): acute respiratory failure secondary to IPF progression (7 patients), pneumonia (7 patients), pneumomediastinum (2 patients), and pneumothorax (1 patient). No case of acute aortic syndrome was recorded. The deaths recorded in IPF patients occurred within 4 years after hospital discharge in the great majority of cases.

On Cox regression analysis, CRP, FVC%, TAPSE/sPAP ratio, and AA indexed to height were independently associated with the primary endpoint (Table 3).

ROC curve analysis revealed that a CRP > 0.37 mg/dL (72% sensitivity, 41% specificity, AUC = 0.54), a FVC < 73.5% (50% sensitivity, 37% specificity, AUC = 0.38), a TAPSE/sPAP ratio < 0.65 mm/mmHg (73% sensitivity, 63% specificity, AUC = 0.73), and an AA diameter indexed to height > 20 mm/m (100% sensitivity, 63% specificity, AUC = 0.78) showed the greatest sensitivity and specificity for predicting the composite endpoint in the whole study population.

**Figure 1:**
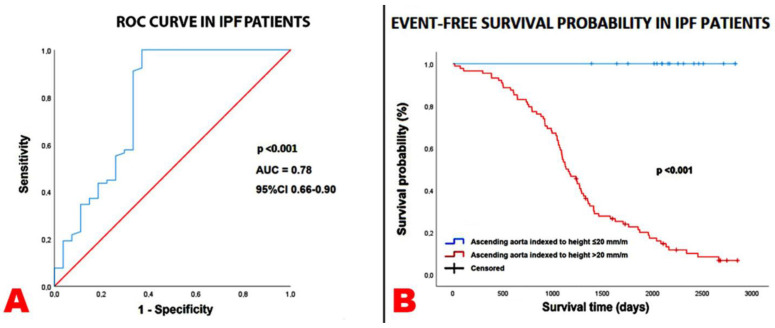
Prognostic ROC curve (**A**) and Kaplan–Meier survival curves (**B**) drawn to compare the rates of the endpoint “all-cause mortality or re-hospitalizations for all causes” in IPF patients, categorized according to ascending aorta indexed to height≤ and >20 mm/m, respectively. AUC, area under the curve; IPF, idiopathic pulmonary fibrosis; ROC, receiver operating characteristics.

Prognostic ROC curves and Kaplan–Meier survival curves were drawn to compare the rates of the composite of “all-cause mortality or re-hospitalization for all causes” over the follow-up period in IPF patients, categorized according to an AA diameter indexed to height ≤ 20 mm/m and >20 mm/m, as depicted in Figure 1A and Figure 1B, respectively.

In the multivariate logistic regression analysis performed to detect the variables independently associated with an AA indexed to height >20 mm/m in the entire cohort of IPF patients, only CRP and LVMi maintained statistical significance (Table 4).

A CRP > 0.4 mg/dL (79% sensitivity, 99% specificity, AUC = 0.93) and an LVMi > 90 g/m^2^ (72% sensitivity, 99% specificity, AUC = 0.83) were the best cut-off values for predicting the secondary endpoint.

### 3.4. Measurement Variability

**Figure 2:**
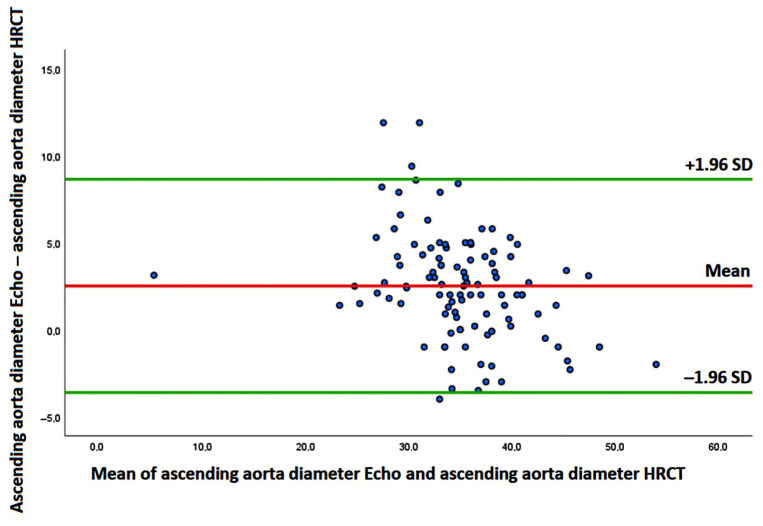
Bland–Altman analysis performed to assess the accuracy and precision of echocardiography-derived ascending aorta diameters compared with non-ECG gated HRCT measurements. ECG, electrocardiogram; HRCT, high-resolution computed tomography.

The Bland–Altman analysis revealed a bias of +2.51 mm (with the 95% limits of agreement ranging from −3.62 to 8.65 mm) for AA estimation, suggesting a general overestimation of the AA diameter by TTE in comparison to non-ECG gated HRCT (Figure 2).

## 4. Discussion

### 4.1. Main Findings of the Study

The present study conducted on a retrospective series of IPF patients without advanced lung disease demonstrated that: (1) compared to matched controls, IPF patients had significantly larger unindexed aortic root and AA diameters and significantly larger aortic root and AA diameters indexed to a height; (2) the AA dilatation was independently associated with the occurrence of the composite of “all-cause mortality or re-hospitalization for all causes” over a mid-term follow-up; (3) the AA diameter indexed to height showed an incremental prognostic value over both the unindexed AA diameter and the AA diameter indexed to BSA; (4) serum levels of CRP and LVMi were independently correlated with AA dilatation in IPF patients; (5) TTE examination was associated with systematic overestimation of AA diameters in comparison to the measurements derived from non-ECG gated HRCT.

In our findings, an AA diameter indexed to height > 20 mm/m at basal evaluation was the best cut-off value for distinguishing between IPF patients with an increased probability of “all-cause mortality or re-hospitalization for all causes” from those with an increased probability of event-free survival, over the follow-up period. Serum CRP levels, FVC%, and the TTE-derived TAPSE/sPAP ratio were other independent prognostic indicators of an increased risk of mortality and adverse clinical events in IPF patients.

### 4.2. Prognostic Role of Ascending Aorta Dilatation

During the last two decades, a few studies have demonstrated the prognostic role of aortic root and AA size in various study populations. Gardin J.M. et al. [11], in a cohort of patients aged ≥ 65 years without clinical CV disease at baseline, found that aortic root dilatation was predictive of congestive heart failure, stroke, CV disease mortality, and all-cause mortality. Lai C.L. et al. [12], in the Chin-Shan Community Cardiovascular Cohort study, found a significant association between aortic root dimension and all-cause death in adults < 65 years. Cuspidi C. et al. [13], in the PAMELA study, demonstrated that the aortic root diameter indexed to height was predictive of nonfatal and fatal CV events among middle-aged individuals, showing an incremental prognostic value over the absolute aortic root diameter and the aortic root diameter indexed to BSA. Kamimura D. et al. [14], in the Jackson Heart Study, found that a greater proximal aortic diameter was associated with an increased risk of CV events in a community-based cohort of blacks. Canciello G. et al. [15] and Leone D. et al. [16] demonstrated that AA dilatation was associated with a greater risk of CV events in hypertensive patients, regardless of LV hypertrophy and other confounders.

Consistent with the PAMELA study’s results, in our findings, the AA diameter indexed to height was the best indicator of poor outcomes in IPF patients. Our results are also in alignment with the recent evidence that indexing aortic dimensions to patient height may allow incremental prognostic information for evaluating the risk of natural complications in patients with AA dilatation [33]. Conversely, the weight might not contribute substantially to aortic size and growth.

The present study also confirmed the usefulness of the TAPSE/sPAP ratio for providing a prognostic risk stratification of IPF patients, as previously demonstrated by our study group [34]. This simple noninvasive index of the RV-PA coupling measures the relationship between RV contractility and RV afterload. The TAPSE/sPAP ratio impairment with consequent RV-PA uncoupling may be induced by a pre-capillary component, resulting from fibrotic destruction of lung parenchyma, leading to hypoxic vasoconstriction and a loss of vascular bed density, and a post-capillary component favored by increased arterial stiffness, moderate diastolic dysfunction, and left-sided heart chambers stiffness.

### 4.3. Pathophysiological Mechanisms of Ascending Aorta Dilatation in IPF Patients

**Figure 3:**
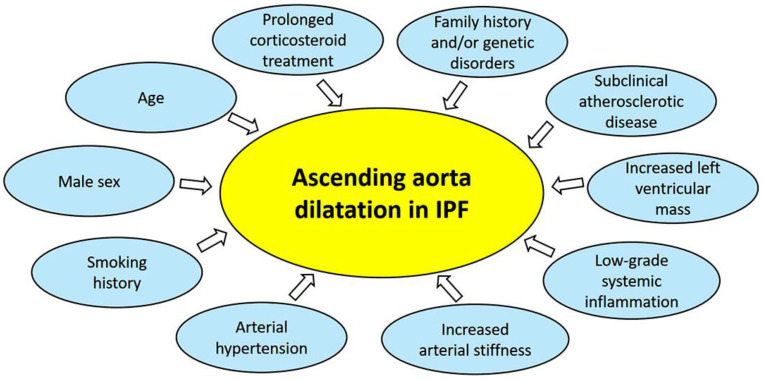
Main determinants of ascending aorta dilatation in IPF patients. IPF, idiopathic pulmonary fibrosis.

A number of factors may synergically contribute to the AA dilatation in IPF patients (Figure 3).

Firstly, it is important to consider that the profile of IPF patients is similar to the general profile of patients at higher risk for CV disease [35], due to the high prevalence of the most common CV risk factors, particularly older age, male sex, smoking history and hypertension, detected in these patients. AA dilatation is a common finding in arterial hypertension, affecting about 15% of hypertensive patients, and has been recently associated with a greater risk of CV events [16].

Secondly, it has been reported that there is an association between poorer pulmonary function and higher arterial stiffness, likely mediated by circulating inflammatory mediators, such as CRP and IL-6 [36]. Given the positive correlation between arterial stiffness and aortic size demonstrated in individuals without overt heart disease [37], in hypertensive patients [38], and in patients affected by type 2 diabetes [39], it is likely that an increased arterial stiffness, frequently observed in IPF patients [19], may have an important role in inducing the AA dilatation in IPF patients.

A low-grade systemic inflammation, which is commonly detected in IPF patients [18,40,41], might contribute to or trigger the process of remodeling within vascular walls of the aorta, with peculiar involvement of the media and adventitia, leading to media atrophy, thinning, and weakening of the aortic wall, and ultimately aortic dilatation [42].

Moreover, as observed in young individuals [43] and in patients with essential hypertension [44], the increase in aortic diameters detected in IPF patients may be related to the increased left ventricular mass. Indeed, LV remodeling is strongly associated with aortic wall remodeling: LV wall thickness, LVMi, and diastolic dysfunction may all contribute to an increase in aortic diameter.

Similarly to what is described in the general population [45], subclinical atherosclerotic disease, particularly CAC score, could be another possible determinant of increased AA diameter in IPF patients.

Aortic dilatation may also be promoted by prolonged corticosteroid treatment, which has been associated with aortic wall fragility due to its negative effect on collagen formation and connective tissue strength [46,47].

Finally, it is not possible to exclude that a number of cases of AA dilatation detected in IPF patients may be related to positive family history and/or genetic disorders, including Marfan syndrome, Ehlers–Danlos syndrome, Turner’s syndrome, and bicuspid aortic valve [48].

In the present study, among the several parameters included in the logistic regression analysis performed to identify the independent predictors of AA dilatation in IPF patients (age, male sex, BSA, hypertension, smoking, CRP, FVC, LVMi, CAC score, and corticosteroid treatment), only serum CRP levels and LVMi showed an incremental prognostic value over the other variables. Our results would confirm that, in IPF patients, LV remodeling and aortic wall remodeling are strongly correlated and that a low-grade systemic inflammation might also affect the AA wall.

### 4.4. Implications for Clinical Practice

In light of our findings, the assessment of aortic diameters might help pulmonologists to optimize the prognostic risk stratification of IPF patients. Notably, IPF patients with AA diameter indexed to height > 20 mm/m at basal echocardiographic evaluation should be considered at high risk of major adverse CV events over a mid-term follow-up period. Due to their beneficial effect in attenuating AA dilation [49,50], the prescription of beta blockers and statins, commonly underutilized in IPF patients [51], should be implemented in clinical practice, especially for those patients with an AA diameter indexed to height > 20 mm/m. The initiation and/or up-titration of angiotensin-converting enzyme (ACE) inhibitors or angiotensin receptor blockers (ARBs) may also protect against further AA dilatation, thus preventing the occurrence of acute aortic syndromes. As observed in hypertensive patients, early cardioprotective treatment could induce a reduction in left ventricular mass [52], thus improving CV prognosis.

The present study also confirmed the accuracy and reliability of conventional TTE for measuring the AA diameters in IPF patients. By providing a systematic overestimation of the real aortic size, the “leading edge-to-leading edge” convention, strongly encouraged by international guidelines [26], is more effective than the “inner edge-to-inner edge” convention in identifying early those patients at increased risk of adverse CV events and/or life-threatening complications, such as aortic dissection.

### 4.5. Study Limitations

The main limitations of the present study were its monocentric design, its retrospective nature, and the limited number of IPF patients analyzed. However, the sample size calculation for survival analysis justified the total number of IPF patients included in the study. In addition, several traditional and innovative echocardiographic parameters, such as stroke volume, arterial elastance, and left atrial strain during the reservoir phase, were not included in our evaluation, as they were not measured in all patients at the basal echocardiographic examination. These parameters would have allowed us to obtain further information about the mechanisms underpinning AA dilatation in IPF patients. Finally, the aortic diameters were assessed by a non-ECG-gated HRCT, rather than computed tomography angiography; therefore, the aortic diameters, even if measured in all IPF patients, could not always be obtained at end-diastole in all cases.

## 5. Conclusions

AA dilatation is predictive of poor outcome in IPF patients without advanced lung disease, over a mid-term follow-up.

The AA diameter indexed to height might improve the prognostic risk stratification of IPF patients, and its dilatation may suggest the early initiation and/or up-titration of cardioprotective drugs.

Further multicentric prospective studies are needed to confirm our findings.

## Figures and Tables

**Table 1:** Clinical characteristics of IPF patients and controls at basal evaluation.

Clinical Variables	IPF Patients (n = 105)	Controls (n = 102)	*p*-Value
**Demographics and anthropometrics**			
Age (yrs)	76.3 ± 6.8	76.4 ± 11.4	0.94
Male sex (%)	82 (78.1)	72 (70.6)	0.22
Height (cm)	166.0 ± 7.7	164.5 ± 9.3	0.21
Weight (Kg)	73.7 ± 13.3	70.7 ± 13.6	0.11
BSA (m^2^)	1.86 ± 0.18	1.85 ± 0.21	0.71
BMI (Kg/m^2^)	26.6 ± 3.5	26.3 ± 4.0	0.56
Yrs from IPF diagnosis	3.7 ± 1.9	/	
**Cardiovascular risk factors**			
Smoking history (%)	86 (81.9)	74 (72.5)	0.11
Hypertension (%)	55 (52.4)	60 (58.8)	0.35
Type 2 diabetes mellitus (%)	34 (32.4)	30 (29.4)	0.64
Dyslipidemia (%)	52 (49.5)	40 (39.2)	0.13
**Atherosclerotic disease burden**			
≥50% carotid artery stenosis (%)	34 (32.4)	19 (18.6)	**0.02**
Coronary artery calcification on HRCT (%)	41 (39.0)	25 (24.5)	**0.02**
Lower extremity peripheral artery disease (%)	12 (11.4)	4 (3.9)	**0.04**
Polidistrectual vasculopathy (%)	24 (22.8)	8 (7.8)	**0.003**
**Cardiovascular disease burden**			
History of CAD (previous PCI/CABG) (%)	23 (21.9)	17 (16.7)	0.34
Previous stroke/TIA (%)	7 (6.7)	12 (11.8)	0.20
**Non-cardiovascular comorbidities**			
Cancers (%)	19 (18.1)	13 (12.7)	0.29
COPD (%)	19 (18.1)	12 (11.8)	0.20
OSAS (%)	9 (8.6)	6 (5.9)	0.45
GERD (%)	24 (22.8)	15 (14.7)	0.13
Hypothyroidism (%)	11 (10.5)	6 (5.9)	0.23
Mixed anxiety–depressive disorder (%)	9 (8.6)	7 (6.9)	0.64
**Blood tests**			
Serum hemoglobin (g/dL)	14.0 ± 1.7	13.7 ± 1.9	0.23
eGFR (mL/min/m^2^)	80.7 ± 17.0	78.0 ± 17.9	0.27
Serum glucose (mg/dL)	115.5 ± 20.6	110.4 ± 33.4	0.19
Serum NT-proBNP (pg/mL)	404.2 ± 1247.2	631.1 ± 1385.0	0.22
Serum CRP (mg/dL)	1.7 ± 2.7	0.9 ± 2.1	**0.02**
Serum LDL cholesterol (mg/dL)	115.1 ± 33.0	107.6 ± 36.4	0.12
**Cardioprotective treatment**			
Antiplatelets (%)	45 (42.8)	50 (49.0)	0.37
Anticoagulants (%)	12 (11.4)	10 (9.8)	0.70
ACEi-ARBs (%)	39 (37.1)	48 (47.0)	0.15
Calcium channel blockers (%)	21 (20.0)	30 (29.4)	0.12
Beta blockers (%)	25 (23.8)	42 (41.2)	**0.007**
Diuretics (%)	28 (26.7)	24 (23.5)	0.60
Statins (%)	22 (20.9)	35 (34.3)	**0.03**
Antidiabetic drugs (%)	28 (26.7)	25 (24.5)	0.72
Proton pump inhibitors (%)	22 (20.9)	13 (12.7)	0.11
**Respiratory treatment**			
Oxygen therapy (%)	55 (52.4)	/	
Oral corticosteroids (%)	38 (36.2)	/	
Inhalation therapy (%)	11 (10.5)	/	
Pirfenidone (%)	43 (40.9)	/	
Nintedanib (%)	55 (52.4)	/	

Data are expressed as mean ± SD or as number (percentage). Significant *p*-values are in bold. ACEIs, angiotensin-converting enzyme inhibitors; ARBs, angiotensin II receptor blockers; BMI, body mass index; BSA, body surface area; CAD, coronary artery disease; CABG, coronary artery bypass graft; COPD, chronic obstructive pulmonary disease; CRP, C-reactive protein; eGFR, estimated glomerular filtration rate; GERD, gastroesophageal reflux disease; HRCT, high resolution computed tomography; IPF, idiopathic pulmonary fibrosis; LDL, low-density lipoprotein; NT-proBNP, N-Terminal pro-B-Type Natriuretic Peptide; OSAS, Obstructive Sleep Apnea Syndrome; PCI, percutaneous coronary intervention; SD, standard deviation; TIA, transient ischemic attack.

**Table 2:** Baseline instrumental variables detected in the two study groups.

Instrumental Parameters	IPF Patients (n = 105)	Controls (n = 102)	*p*-Value
**Radiological findings**			
Definite UIP (%)	63 (60.0)	/	/
Probable UIP (%)	26 (24.8)	/	/
Indeterminate pattern (%)	16 (15.2)	/	/
CAC score (HU)	698.9 ± 879.8	/	/
**Spirometry parameters**			
FVC (L)	2.6 ± 0.6	/	/
FVC (%)	77.6 ± 16.7	/	/
FEV1 (L)	2.1 ± 0.5	/	/
FEV1 (%)	82.4 ± 16.1	/	/
FEV1/FVC ratio	0.8 ± 0.1	/	/
TLC (L)	4.8 ± 1.1	/	/
TLC (%)	76.4 ± 16.7	/	/
DLCO (mL/min/mmHg)	11.4 ± 4.0	/	/
DLCO (%)	47.9 ± 16.0	/	/
Restrictive pattern (%)	70 (66.7)	/	/
ΔSaO_2_ (%)	6.6 ± 4.2	/	/
6MWT (m)	399.3 ± 110.6	/	/
**ECG variables**			
Heart rate (bpm)	74.9 ± 15.0	74.1 ± 12.2	0.67
AF (%)	12 (11.4)	10 (9.8)	0.70
Intraventricular delay (%)	21 (20.0)	25 (24.5)	0.43
**EchoDoppler parameters**			
LVEDD (mm)	46.5 ± 5.7	47.0 ± 6.2	0.55
RWT	0.43 ± 0.06	0.43 ± 0.07	>0.99
LVMi (g/m^2^)	97.5 ± 23.3	102.6 ± 29.2	0.16
Normal LV geometric pattern (%)	36 (34.3)	30 (29.4)	0.45
LV concentric remodeling (%)	48 (45.7)	40 (39.2)	0.34
LV concentric hypertrophy (%)	10 (9.5)	16 (15.7)	0.18
LV eccentric hypertrophy (%)	11 (10.5)	16 (15.7)	0.26
LVEDVi (mL/m^2^)	40.3 ± 11.8	38.7 ± 13.5	0.36
LVESVi (mL/m^2^)	15.8 ± 8.2	14.7 ± 10.3	0.39
LVEF (%)	61.9 ± 9.1	63.8 ± 9.4	0.14
E/A ratio	0.78 ± 0.18	0.75 ± 0.44	0.52
E/average e’ ratio	14.0 ± 4.5	11.9 ± 4.9	**0.001**
LAVi (mL/m^2^)	33.6 ± 10.9	34.1 ± 13.7	0.77
More than mild MR (%)	12 (11.4)	11 (10.8)	0.88
More than mild AR (%)	11 (10.5)	8 (7.8)	0.51
More than mild TR (%)	24 (22.8)	7 (6.9)	**0.001**
RVIT (mm)	33.1 ± 6.6	28.1 ± 4.3	**<0.001**
RV/LV basal diameter ratio	0.77 ± 0.23	0.70 ± 0.19	**0.02**
TAPSE (mm)	22.0 ± 4.7	22.9 ± 3.7	0.13
TRV (m/s)	3.3 ± 2.7	2.6 ± 0.3	**0.009**
IVC (mm)	19.7 ± 4.8	17.9 ± 3.8	**0.003**
sPAP (mmHg)	42.0 ± 13.3	27.7 ± 6.3	**<0.001**
TAPSE/sPAP (mm/mmHg)	0.57 ± 0.24	0.86 ± 0.22	**<0.001**
Unindexed aortic root (mm)	36.4 ± 3.8	34.9 ± 4.1	**0.007**
Aortic root indexed to BSA (mm/m^2^)	19.6 ± 2.2	19.3 ± 2.4	0.35
Aortic root indexed to height (mm/m)	22.0 ± 2.3	21.2 ± 2.1	**0.009**
Unindexed ascending aorta (mm)	36.6 ± 4.9	35.0 ± 3.9	**0.01**
Ascending aorta indexed to BSA (mm/m^2^)	19.8 ± 3.0	19.4 ± 2.7	0.31
Ascending aorta indexed to height (mm/m)	22.1 ± 2.9	21.3 ± 2.4	**0.03**

Data are expressed as mean ± SD or as number (percentage). Significant *p*-values are in bold. A 6MWT, six-minute walking test; ΔSaO2, absolute difference between peak exercise and rest oxygen saturation; AR, aortic regurgitation; BSA, body surface area; CAC, coronary artery calcification; DLCO, diffusing capacity of the lung for carbon monoxide; ECG, electrocardiogram; FEV1, forced expiratory volume in 1 s; FVC, forced vital capacity; HRCT, high resolution computed tomography; HU, Hounsfield unit; IPF, idiopathic pulmonary fibrosis; IVC, inferior vena cava; LAVi, left atrial volume index; LV, left ventricular; LVEDD, left ventricular end-diastolic diameter; LVEDVi, left ventricular end-diastolic volume index; LVEF, left ventricular ejection fraction; LVESVi, left ventricular end-systolic volume index; LVMi, left ventricular mass index; MR, mitral regurgitation; RV, right ventricular; RVIT, right ventricular inflow tract; RWT, relative wall thickness; SD, standard deviation; sPAP, systolic pulmonary artery pressure; TAPSE, tricuspid annular plane systolic excursion; TLC, total lung capacity; TR, tricuspid regurgitation; TRV, tricuspid regurgitation velocity; TTE, transthoracic echocardiography; UIP, usual interstitial pneumonia.

**Table 3:** Univariate and multivariate Cox regression analyses performed to identify the variables independently associated with the composite of “all-cause mortality plus re-hospitalization for all causes” in the whole cohort of IPF patients, over a medium-term follow-up.

	Univariate Cox Regression Analysis			Multivariate Cox Regression Analysis		
Variables	HR	95% CI	*p*-Value	HR	95% CI	*p*-Value
Age (yrs)	1.02	0.98–1.05	0.32			
Male sex	1.33	0.77–2.31	0.31			
Smoking	1.15	0.65–2.02	0.64			
CRP (mg/dL)	1.12	1.05–1.20	**<0.001**	1.09	1.01–1.18	**0.03**
FVC (%)	0.98	0.96–0.99	**0.002**	0.98	0.97–0.99	**0.02**
Definite UIP pattern	1.20	0.88–1.64	0.24			
LVEF (%)	0.97	0.94–0.99	**0.03**	0.98	0.95–1.00	0.11
TAPSE/sPAP ratio (mm/mmHg)	0.10	0.03–0.34	**<0.001**	0.23	0.07–0.76	**0.02**
Unindexed ascending aorta diameter	1.01	0.96–1.06	0.83			
Ascending aorta diameter indexed to BSA (mm/m^2^)	1.00	0.89–1.11	0.39			
Ascending aorta diameter indexed to height (mm/m)	1.18	1.09–1.27	**<0.001**	1.15	1.06–1.25	**<0.001**
CAC score (HU)	1.00	0.97–1.03	0.98			
Beta blocker treatment	0.79	0.50–1.26	0.32			

Significant *p*-values are in bold. BSA, body surface area; CAC, coronary artery calcification; CRP, C-reactive protein; FVC, forced vital capacity; HU, Hounsfield unit; LVEF, left ventricular ejection fraction; sPAP, systolic pulmonary artery pressure; TAPSE, tricuspid annular plane systolic excursion; UIP, usual interstitial pneumonia.

**Table 4:** Univariate and multivariate logistic regression analyses performed to evaluate the parameters independently associated with an ascending aorta indexed to height > 20 mm/m in the entire cohort of IPF patients.

	Univariate Logistic Regression Analysis			Multivariate Logistic Regression Analysis		
Variables	OR	95% CI	*p*-Value	OR	95% CI	*p*-Value
Age (yrs)	1.03	0.95–1.11	0.43			
Male sex	1.62	0.51–5.19	0.42			
BSA (m^2^)	1.66	0.10–30.5	0.73			
Hypertension	1.71	0.59–4.91	0.32			
Smoking	2.20	0.67–7.23	0.19			
CRP (mg/dL) x 0.1 U increase	2.00	1.30–3.06	**0.001**	1.87	1.21–2.89	**0.005**
FVC (%)	0.96	0.93–0.99	**0.03**	0.98	0.93–1.03	0.36
Definite UIP pattern	1.05	0.52–2.13	0.88			
LVMi (g/m^2^)	1.08	1.04–1.13	**<0.001**	1.13	1.04–1.24	**0.006**
CAC score (HU)	1.02	0.95–1.09	0.52			
Oral corticosteroids	1.77	0.53–5.89	0.35			

Significant *p*-values are in bold. BSA, body surface area; CAC, coronary artery calcification; CRP, C-reactive protein; FVC, forced vital capacity; HU, Hounsfield unit; LVMi, left ventricular mass index; UIP, usual interstitial pneumonia.

## Data Availability

Data extracted from included studies will be publicly available on Zenodo (https://zenodo.orgaccessed on 31 October 2024).
